# LONELINESS AND COGNITIVE FUNCTION IN OLDER ADULTS WITHOUT DEMENTIA: A SYSTEMATIC REVIEW

**DOI:** 10.1093/geroni/igac059.2611

**Published:** 2022-12-20

**Authors:** Karra Harrington, Shradha Vasan, Jee eun Kang, Martin Sliwinski, Michelle Lim

**Affiliations:** The Pennsylvania State University, University Park, Pennsylvania, United States; Swinburne University of Technology, Hawthorn, Victoria, Australia; The Pennsylvania State University, University Park, Pennsylvania, United States; The Pennsylvania State University, University Park, Pennsylvania, United States; Swinburne University of Technology, Hawthorn, Victoria, Australia

## Abstract

Loneliness has consistently been associated with dementia risk. An important precursor to dementia is cognitive decline, which can begin decades prior to clinical diagnosis of dementia. Therefore, understanding about the relationship between loneliness and cognitive function in healthy older adults may inform our understanding of how loneliness contributes to dementia risk. The aim of this systematic review was to identify the extent to which loneliness affects cognition in older adults who do not have dementia. A systematic search of five databases (PubMed, PsycNET, Web of Science, EBSCOhost, Scopus) from inception to August 31st 2021 was completed, including search terms related to loneliness, aging, and cognition. A total of 4,302 unique articles were screened for inclusion, resulting in 16 studies that met full criteria (six cross-sectional and ten longitudinal). Three of the six (50%) cross-sectional studies reported significant negative associations between loneliness and cognitive function, while six of the ten (60%) longitudinal studies reported that loneliness was associated with greater cognitive decline over time. We did not find a significant relationship between loneliness and cognitive function in the rest of the studies. There was substantial variation across studies in the measures of loneliness and cognitive function. Furthermore, many studies relied on cognitive screening tools to identify cognitive outcomes, which may not be sensitive to subtle cognitive changes that precede dementia. Future studies should consider using validated and sensitive measures of loneliness and cognitive function, and examining these relationships prospectively, in order to, assess these relationships in a more robust way.

